# Factors contributing to airborne particle dispersal in the operating room

**DOI:** 10.1186/s12893-017-0275-1

**Published:** 2017-07-06

**Authors:** Chieko Noguchi, Hironobu Koseki, Hidehiko Horiuchi, Akihiko Yonekura, Masato Tomita, Takashi Higuchi, Shinya Sunagawa, Makoto Osaki

**Affiliations:** 1 Department of Orthopedic Surgery, Nagasaki University Graduate School of Biomedical Sciences, 1-7-1 Sakamoto, Nagasaki, 852-8501 Japan; 2 Department of Locomotive Rehabilitation Science, Unit of Rehabilitation sciences, Nagasaki University Graduate School of Biomedical Sciences, 1-7-1 Sakamoto, Nagasaki, 852-8520 Japan

**Keywords:** Surgery, Airborne particle, Surgical-site infection, Intraoperative action

## Abstract

**Background:**

Surgical-site infections due to intraoperative contamination are chiefly ascribable to airborne particles carrying microorganisms. The purpose of this study is to identify the actions that increase the number of airborne particles in the operating room.

**Methods:**

Two surgeons and two surgical nurses performed three patterns of physical movements to mimic intraoperative actions, such as preparing the instrument table, gowning and donning/doffing gloves, and preparing for total knee arthroplasty. The generation and behavior of airborne particles were filmed using a fine particle visualization system, and the number of airborne particles in 2.83 m^3^ of air was counted using a laser particle counter. Each action was repeated five times, and the particle measurements were evaluated through one-way analysis of variance multiple comparison tests followed by Tukey–Kramer and Bonferroni–Dunn multiple comparison tests for post hoc analysis. Statistical significance was defined as a *P* value ≤ .01.

**Results:**

A large number of airborne particles were observed while unfolding the surgical gown, removing gloves, and putting the arms through the sleeves of the gown. Although numerous airborne particles were observed while applying the stockinet and putting on large drapes for preparation of total knee arthroplasty, fewer particles (0.3–2.0 μm in size) were detected at the level of the operating table under laminar airflow compared to actions performed in a non-ventilated preoperative room (*P* < .01).

**Conclusions:**

The results of this study suggest that surgical staff should avoid unnecessary actions that produce a large number of airborne particles near a sterile area and that laminar airflow has the potential to reduce the incidence of bacterial contamination.

## Background

The Centers for Disease Control and Prevention’s National Nosocomial Infection Surveillance (NNIS) system reported 15,523 surgical-site infections (SSIs) following 593,344 operations between 1986 and 1996, and 77% of the deaths following complications from surgery were reported to be related to SSI [1]. Especially in the field of orthopedics, SSI after prosthetic arthroplasty is a devastating complication because treating the infection requires several procedures at considerable expense. The incidence of SSI in the United States after primary total hip arthroplasty (THA) is 0.88% and on the rise, whereas the infection rate for revision THA is more than double that for primary procedures [2]. Though SSIs are multi-factorial in origin and include both patient- and procedure-specific factors, airborne infection is thought to be one of the major sources of exogenous contaminating bacteria [3–5]. During surgical procedures, bacteria-laden airborne particles, including textile fibers, dust particles, skin fragments, and respiratory aerosols, may settle on surgical instruments or directly enter the surgical site, resulting in SSI [6–9]. Hansen et al. noted that bacterial counts were lower in environments with fewer airborne particles, and that the number of particles larger than 5 μm was closely correlated with bacterial concentration [10]. Campbell et al. reported that a decreased turnover of operating staff resulted in lower rates of SSI [11]. Other studies have demonstrated that 80%–90% of pathogenic bacteria detected from surgical wounds were related to airborne particles in the operating room [12] and that airborne skin scales can act as vectors for pathogenic microorganisms to infect the surgical wound [13]. The Healthcare Infection Control Practices Advisory Committee guidelines for the prevention of SSI published in 1999 recommended to “consider performing orthopedic implant operations in operating rooms supplied with ultraclean air” and classified this recommendation as category II (suggested for implementation and supported by suggestive clinical or epidemiological studies or theoretical rationale) [1]. Thus, surgical-site contamination by airborne microorganisms plays a central role in the exogenous pathogenesis of SSIs, and controlling and minimizing airborne particles in the operating room deserves close attention to protect patients against exogenous infection caused by airborne bacteria.

Non-woven fabric, widely used for surgical drapes, gowns, and hoods, is thought to be one of the major origins of airborne particles in the operating room. There is a high level of activity involving fabrics during preoperative preparation of a patient, resulting in the dispersal of a large number of airborne particles [14]. Textile fibers from non-woven fabric may migrate to or come in contact with unsterile areas, such as the walls, floor, and human skin. Therefore, a greater number of particles produced from non-woven fabric increases the chances of airborne particles being contaminated with bacteria. Although any action in the operating room can produce particles, the degree to which these actions generate particles remains unclear, and the dispersal conditions of airborne particles during preoperative procedures has not yet been visualized. To prevent SSIs, operating staff including surgeons must understand the situations that are at high risk for producing airborne particles in the operating room.

The aim of this study is to investigate and quantify the dispersion and distribution of airborne particles due to actions in the operating room.

## Methods

### Experimental design

All surgical drapes and garments used in this study were made from generally used spunlaced non-woven fabric that consisted of 45% wood pulp and 55% polyester pulp. Spunlacing is a technique used to give a web of fibers sufficient cohesion by mechanical bonding, while the paper-making technique allows the production of a web where the fibers are consolidated by hydroentanglement. The water jet pressure was up to 100 bar. After removal of water by suction, the non-woven fabric was air dried (180 °C). The surface density of the non-woven fabric was 80 g/m^2^. The authors performed the following three patterns of physical movements in the present study to mimic some of the intraoperative actions that take place during major orthopedic surgery.

#### Preparing instrument table

Step 1: an assistant holds and opens a sterilized package. Step 2: the operating room nurse removes the folded surgical drape (DEF-58-T®, hopes Co. Ltd., Hokkaido, Japan) from the package and unfolds it in front of his or her chest. Step 3: the nurse slowly spreads the drape on an instrument Table (1 m high, 80 cm wide, and 50 cm deep).

#### Gowning and donning/doffing gloves

Step 1: an assistant holds and opens a sterilized package. Step 2: the surgeon removes the folded surgical gown (JG-100®, hopes Co. Ltd.) made from spunlaced non-woven fabric from the package and unfolds it in front of his or her chest. Step 3: a circulating nurse helps the surgeon put on the surgical gown according to the traditional closed gowning technique. Step 4: the surgeon puts on and takes off latex powdered surgical gloves (Tradition®, Medline International Japan, Tokyo, Japan). Procedures 1 and 2 were performed in a non-ventilated preoperative room.

#### Preparation for total knee arthroplasty (TKA)

Step 1: one of the co-authors acting as a patient is laid on the operating table and positioned correctly under laminar airflow (LAF) in a bio-clean room (ISO class 7 criterion; Fed. Standard class 10,000) with a high-efficacy particulate air (HEPA) filter. The settings for LAF were: wind velocity, 0.44 m/s; room temperature, 21.9 °C; and humidity, 32.4%. Step 2: after all surgeons were gowned with Sterishield Togas and T4 helmets (Stryker Instruments, Kalamazoo, MI, USA), one surgeon lifted the patient’s left leg. Another surgeon applied a stockinet and wrapped the leg with an elastic bandage. Step 3: one surgeon fit three hydrophobic drapes (RH-33®, hopes Co. Ltd.) around the patient’s thigh, and then the surgeons passed the patient’s leg through a large, holed drape (RH-710EFC90®, hopes Co. Ltd.). Step 4: a surgeon cut and removed the piece of stockinet from around the surgical site and covered the patient’s leg with an iodine-impregnated plastic film.

The generation and behavior of airborne particles were filmed using a fine particle visualization system (Shin-Nihon Air Technologies Co. Ltd., Tokyo, Japan) with a green laser apparatus. After making a uniform laser sheet, light reflected from airborne particles was filmed using a highly sensitive camera with an interference filter. The number of airborne particles in 2.83 m^3^ of air was counted using a laser particle counter (KC-52®, RION, Tokyo, Japan), and the mean value was taken as the measured value. Sampling was performed at 1.1 m above floor level, simulating the height of the operating table. The sampling tube (6 mm internal diameter) was attached to the air intake port of the particle counter, and the measurement interval was set to 1 min (2.83 m^3^). Particles were separated into four categories based on their size (0.3–0.5 μm, 0.5–1.0 μm, 1.0–2.0 μm, and 2.0–5.0 μm).

### Statistical analysis

Each established intraoperative action was repeated five times, and the particle measurements were compiled for statistical analysis, which included one-way analysis of variance multiple comparison tests followed by Tukey–Kramer and Bonferroni–Dunn multiple comparison tests for post hoc analysis, using SPSS version 22.0 (SPSS, Chicago, IL, USA). Values are expressed as means ± standard deviations. Statistical significance was defined as a *P* value ≤ .01.

## Results

### Preparing instrument table

The fine particle visualization system showed that many particles were dispersed in the antero-inferior direction while the operating room nurse unfolded a surgical drape. The mean number of airborne particles for every action is shown in Table 1. Most of the particles detected were 0.3–0.5 μm in size.

**Table 1:** Mean number and standard deviation of airborne particles (particles/2.83 m^3^)

Particle size category (μm)	0.3–0.5	0.6–1.0	1.1–2.0	2.1–5.0
Preparing instrument table	16,826 (509.1)	1423 (33.9)	187 (7.8)	128 (14.8)
Gowning and donning/doffing gloves	18,075 (4202.7)	1589 (344.7)	232 (49.6)	173 (31.4)
Preparation for TKA	1207 (125.9)^§*^	202 (15.6)^§*^	66 (2.8)^*^	109 (0.7)

^§^: *P* < .01 compared to the actions of preparing the instrument table

^*^: *P* < .01 compared to the actions of gowning and donning/doffing gloves

### Gowning and donning/doffing gloves

**Fig. 1:**
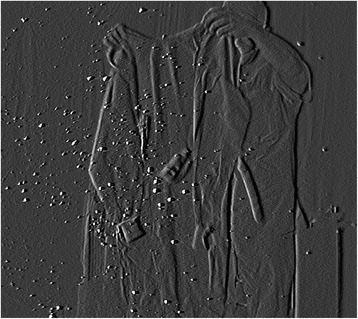
Unfolding the surgical gown. The dispersal of reflective airborne particles (*bright dots*) could be observed with a fine particle visualization system

**Fig. 2:**
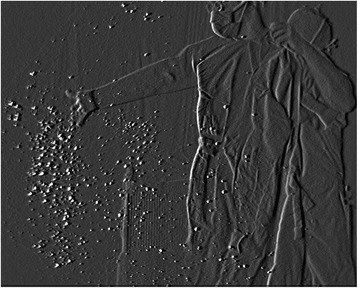
Putting arms through the sleeves. A large number of particles burst from the cuffs of the gown

**Fig. 3:**
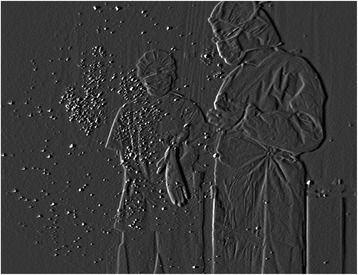
Removing surgical gloves. Particles including powder, sweat, and skin fragments dispersed and floated in the air

Similar to when unfolding the drape, many particles were dispersed in the antero-inferior direction while the surgeon unfolded a surgical gown (Fig. 1). Notably, particles burst from the cuffs or collar of the gown the moment the arms were put through the sleeves and the tail of the gown was stretched (Fig. 2). Moreover, a lot of small airborne particles, which were thought to be powder, sweat, and skin fragments, were observed when the surgeon removed the surgical gloves (Fig. 3). The mean number of airborne particles during gowning and donning/doffing surgical gloves was similar to that during preparation of the instrument table.

### Preparation for TKA

**Fig. 4:**
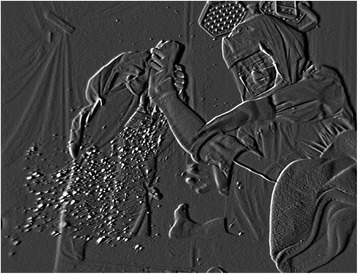
Applying stockinet. One surgeon lifted the left leg of an author acting as a patient and another surgeon applied a stockinet. Many particles were produced around the patient’s leg and then migrated downward slowly under laminar airflow

Before any actions, airborne particles in the bio-clean room drifted downward slowly under LAF. The actions of applying a rolled stockinet (Fig. 4), and cutting the elastic bandage generated a lot of airborne particles. Additionally, when placing the large drape with a hole in the center over the leg, many particles were generated under the drape as it rubbed the stockinet. However, most of the particles drifted downward slowly due to the LAF. As a result, the counts in the bio-clean room for particles (0.3–1.0 μm in size) were significantly lower compared to those when preparing the instrument table or when gowning and donning/doffing gloves (*P* < .01). The counts for particles (1.1–2.0 μm in size) were also significantly lower than those when gowning and donning/doffing gloves (*P* < .01).

## Discussion

The microorganism most often responsible for SSIs is *Staphylococcus aureus*, which can adhere to particles. Airborne transmission has been implicated in nosocomial outbreaks of methicillin-resistant *Staphylococcus aureus* (MRSA) [15]. Because MRSA range from 0.8 to 1.0 μm in diameter, it is anticipated that not only larger sized airborne particles but also aggregates of smaller sized airborne particles held together by static electricity can be laden with pathogenic bacteria. Surgical drapes and garments are thought to be two of the major origins of airborne textile fiber particles. This is one reason why the material of surgical drapes and garments has been switched from cotton to non-woven fabric [16]. Cotton can generate many textile fiber particles, and woven cotton has interlacing gaps ranging from 7 to 50 μm in diameter that can easily pass bacteria-laden airborne particles or skin fragments from medical staff. Even non-woven fabrics, however, may generate many textile fiber particles depending on the action of the wearer in the operating room. Therefore, prediction and reduction of particle dispersion and distribution from non-woven fabrics are key to lowering the risk of contamination by airborne microorganisms.

In our study, a high number of dispersed airborne particles were observed when unfolding the drape and surgical gown. Since the drape and surgical gown were initially sterile, the particles from them are considered to be free of bacteria. However, airborne particles can act as vectors for transmission of bacteria after coming in contact with unsterile areas (e.g. skin, walls, or floor) [4]. Particles settled on an unsterile floor can be easily dispersed by air eddies generated from opening doors and foot traffic. A recent study noted a trend towards lower SSI rates in hospitals with decreased operating room staff turnover [11]. Thus, it is preferable that the actions such as unfolding a drape and surgical gown should be carried out away from the operating and instrument Tables.

A greater number of scattered particles were also seen when removing gloves, putting the arms through the sleeves of the surgical gown, and stretching the tail of the gown. Individuals in the operating room generate many bacteria-laden skin fragments [17, 18], which may migrate from sites of uncovered skin (e.g. neck and face) or through gaps in the material used to make surgical garments [19]. Dharan and Pittet reported that more than half of all infections following clean surgery were caused by the normal skin flora of patients and healthcare workers [20]. Dispersed airborne particles visualized during removal of surgical gloves and during donning a surgical gown in this study are thought to contain many skin fragments and bacteria-laden textile fibers or powders that may cause SSIs. Regarding SSIs and surgical gloves, most of the recommendations focus on the risk of permeability and perforation, and there is no evidence associated with particle dispersion [21–23]. Our findings support a clear practical recommendation—removing gloves and donning a surgical gown should be strictly avoided near the surgical site or sterile instruments.

Moreover, surgeons should pay close attention to minimizing the production of airborne particles while applying or cutting an elastic bandage or stockinet and covering a limb with a holed drape, especially for immunocompromised patients. Our results demonstrated that both an elastic bandage and stockinet made of cotton produce many textile fiber particles when cut, stretched, or even rubbed close to the surgical site. Interestingly, although many particles were observed during preparation for TKA, only a small number of airborne particles were detected at the level of the operating table. The LAF system, which is commonly used in bio-clean rooms [24], creates a homogenous, low-turbulence airflow directly over the operating area through a combination of high airflow rates and HEPA filtration [10]. Laminar airflow with HEPA filters can remove approximately 99.97% of airborne particles larger than 0.3 μm, resulting in minimal air bacterial counts [6, 20]. The fine particle visualization system used in the present study revealed that airborne particles in the operating room drifted downward slowly under LAF. This is why there were fewer particles at the level of the operating table compared to the number of particles detected in the non-ventilated preoperative room. Recently, some publications have questioned whether LAF ventilation confers any benefit and even suggest that postoperative SSI rates may be higher after surgery under LAF conditions compared to conventional operating rooms with turbulent ventilation [25, 26]. The most recent global guidelines from the World Health Organization on the prevention of SSI also suggested that LAF ventilation systems should not be used for patients undergoing total arthroplasty [27]. However, the strength of the recommendation is “conditional level”, and the quality of the evidence is “low to very low”. Moreover, the onset of SSIs is influenced by multiple factors, including the virulence of the bacteria, quality of the patient’s immune defenses, and prophylactic antibiotic therapy. Therefore, although the relationship between LAF systems and SSI rates remains unclear, it can be speculated from our results that LAF can decrease the chances of bacterial air contamination.

Each action investigated in the present study was in preparation for TKA, and not representative of the entire operation. Although surgical-site bacterial counts correlate with airborne bacteria and particle counts [3–5], they have not been demonstrated to correlate directly with the rate of SSIs [4]. The actual relationship among the amount of particles, the incidence of bacterial contamination, and the rate of SSIs was not addressed in this study. The present results, obtained using well-defined environmental conditions, cannot necessarily be translated directly to different settings, i.e. different sized operating rooms or a different number of personnel within the operating room. However, our study simulating some of the intraoperative actions gives surgical staff a clearer picture of the dispersion and distribution of particles that could contaminate the surgical site. Surgical staff should consider carefully measures to minimize the production of airborne particles and decrease particle counts during intraoperative procedures to lower the risk of contamination by airborne microorganisms.

## Conclusions

Fine particle visualization and automatic particle counting revealed that a large number of airborne particles were produced during unfolding the surgical gown, removal of gloves and placing arms through the sleeves of the gowns. Medical staff in the operating room should avoid those actions near sterile areas. Fewer particles were detected at the level of the operating table under laminar airflow, which suggests that laminar airflow has the potential to reduce the incidence of bacterial contamination.

## Abbreviations

HEPA: High-efficacy particulate air
LAF: Laminar airflow
MRSA: Methicillin-resistant *Staphylococcus aureus*
NNIS: National Nosocomial Infection Surveillance
SSIs: Surgical-site infections
THA: Total hip arthroplasty
TKA: Total knee arthroplasty
